# The Digital Divide and Health Disparities in China: Evidence From a National Survey and Policy Implications

**DOI:** 10.2196/jmir.7786

**Published:** 2017-09-11

**Authors:** Y Alicia Hong, Zi Zhou, Ya Fang, Leiyu Shi

**Affiliations:** ^1^ School of Public Health Xiamen University Xiamen China; ^2^ School of Public Health Texas A&M University College Station, TX United States; ^3^ Department of Health Policy and Management Bloomberg School of Public Health Johns Hopkins University Baltimore, MD United States

**Keywords:** digital divide, health disparities, Internet, mobile phone, China

## Abstract

**Background:**

The digital divide persists despite broad accessibility of mobile tools. The relationship between the digital divide and health disparities reflects social status in terms of access to resources and health outcomes; however, data on this relationship are limited from developing countries such as China.

**Objective:**

The aim of this study was to examine the current rates of access to mobile tools (Internet use and mobile phone ownership) among older Chinese individuals (aged ≥45 years), the predictors of access at individual and community levels, and the relationship between access to mobile tools and health outcomes.

**Methods:**

We drew cross-sectional data from a national representative survey, the China Health and Retirement Longitudinal Study (CHARLS), which focused on the older population (aged ≥45 years). We used two-level mixed logistic regression models, controlling for unobserved heterogeneity at the community and individual levels for data analysis. In addition to individual-level socioeconomic status (SES), we included community-level resources such as neighborhood amenities, health care facilities, and community organizations. Health outcomes were measured by self-reported health and absence of disability based on validated scales.

**Results:**

Among the 18,215 participants, 6.51% had used the Internet in the past month, and 83% owned a mobile phone. In the multivariate models, Internet use was strongly associated with SES, rural or urban residence, neighborhood amenities, community resources, and geographic region. Mobile phone ownership was strongly associated with SES and rural/urban residence but not so much with neighborhood amenities and community resources. Internet use was a significant predictor of self-reported health status, and mobile phone ownership was significantly associated with having disability even after controlling for potential confounders at the individual and community levels.

**Conclusions:**

This study is one of the first to examine digital divide and its relationship with health disparities in China. The data showed a significant digital divide in China, especially in the older population. Internet access is still limited to people with higher SES; however, the mobile phone has been adopted by the general population. The digital divide is associated with not only individual SES but also community resources. Future electronic health (eHealth) programs need to consider the accessibility of mobile tools and develop culturally appropriate programs for various social groups.

## Introduction

Over the past decade, global access to mobile technologies such as the Internet and cell phones has dramatically increased. Such access has transformed the way people receive information and communicate with one another; in fact, prior studies have shown that those with access to mobile technologies have better mental health, physical health, and medical decision-making skills [1,2]. Accompanying the “Internet of Things” and the “e-lifestyle” is the persistent digital divide, defined as the gap between those with access to new forms of information technology and those without. [3-5]. Even in the Western countries with high penetration rates of mobile tools, digital inequality remains significant [6-8], and the digital divide strongly correlates with health disparities [9,10].

Literature on the relationship of the digital divide and health disparities suggests that an individual’s lifestyle choices, including the use of mobile technologies, are not autonomous but are instead constrained or enabled by one’s social status and access to economic and other resources [3,4,11-13]. Having access to resources including mobile technologies enables an individual, family, or social group to receive more up-to-date health information, to obtain social support, to adopt healthy behaviors, and to make more informed medical decisions, and therefore, have better health outcomes; this in turn reshapes their socioeconomic status (SES; [3,10,13]). Empirical data corroborated this theory and showed that both the digital divide and health disparities are predicted by older age, low level of education, low income, racial or ethnic minority status, and rural residence [3-13].

Current literature on digital divide and health disparities is limited in three aspects. First, existing studies on predictors of the digital divide and its relationship with health disparities have been limited to individual-level SES factors such as age, race/ethnicity, gender, education, and income; however, community-level factors such as neighborhood characteristics and community resources have rarely been examined. Sociologists have long documented the impact of community on individual health and well-being [14-16]. Second, only a few studies on the digital divide and health disparity have been conducted in older adults, who are often the last group to adopt technology and more likely to face health disparities [8,17,18]. In many countries, the elder population is the fastest growing population but has been left out of the accelerative “e-lifestyle” movement [19-22]. Third, most existing studies on the digital divide and health disparities were conducted in Western countries, with limited data from developing countries, despite the rapid adoption rates of mobile technologies in these countries [23,24]. For example, China is home to a quarter of the world’s population; and as of 2016, China had 731 million Internet users (penetration rate: 53%) and 1.3 billion cell phone users (penetration rate: 95%; [25,26]). The Chinese government has been active in promoting the application of the Internet and mobile phones in health services delivery [27-29]. Studies on the use of mobile tools in special populations could be traced back to more than a decade ago, right from the 2000s [30-32]; recent literature indicates that both the Internet and mobile phones have been used in public health emergency responses [33], infectious disease surveillance [34], teleconsultation [35], and intervention delivery [36,37]. Despite the growing body of literature on the application of mobile tools, to date, no study exists on the digital divide in the Chinese population, especially in the older population.

With the aim to fill gaps in the literature, in this study we used data from the 2011 and 2013 waves of the China Health and Retirement Longitudinal Study (CHARLS) to examine the relationship between the digital divide and health disparities. We aim to answer the following research questions: (1) among the general middle-aged and elderly Chinese (45 years and older), what is the prevalence of access to mobile tools (Internet use and cell phone ownership)?; (2) what are the predictors of Internet use and mobile phone ownership at individual and neighborhood levels?; and (3) what is the relationship between health outcomes and access to mobile tools after controlling for potential confounders?

## Methods

### Data Source

The CHARLS is a nationally representative longitudinal survey of the middle-aged and elderly population (45+ years) in China. As detailed in previous reports [38-40], led by the Peking University in China via collaboration with the Oxford University in the United Kingdom and the University of Southern California in the United States, CHARLS was designed as a part of a set of international longitudinal aging surveys that include the Health and Retirement Study (HRS) in the United States, the Survey of Health and Retirement in Europe (SHARE), and similar longitudinal aging surveys in other countries. Following the protocols of the HRS, the CHARLS main questionnaire comprises seven modules, covering demographics, family background, health status, SES, and environment (community questionnaire and county-level policy questionnaire). All data were collected through face-to-face, computer-aided personal interviews (CAPI; [38-40]).

### Sample Size

The national baseline was conducted from July 2011 to March 2012 and represented people aged 45 years and older, living in 150 counties in 28 provinces across China. A total of 150 county-level units were randomly selected using probability proportional to size (PPS) and stratified by region, urban/rural and county-level gross domestic product (GDP). Within each county-level unit, three village-level units (villages in rural areas and urban communities in urban areas) were randomly selected using PPS as primary sampling units (PSUs). Within each PSU, 80 dwellings were randomly selected from a complete list of dwelling units generated from a mapping or listing operation, using augmented Google Earth maps (Google Inc) along with considerable ground checking. In scenarios with more than one age-eligible household in a dwelling unit, one was randomly selected. From this sample for each PSU, the proportion of households with age-eligible members was determined, as was the proportion of empty residences. From these proportions and an assumed response rate, we selected households from our original PSU frame to obtain a target number of 24 age-eligible households per PSU. Thus, the final household sample size in a PSU depended on the PSU age-eligibility and empty residence rates. In each household, one person aged 45 years or older was randomly chosen as the main respondent, and the individual’s spouse was automatically included. On the basis of this sampling procedure, 1 or 2 individuals in each household were interviewed depending on the marital status of the main respondent. The total sample size was 10,257 households and 10,481 individuals in the 2011 baseline. The sample size for the first follow-up in 2013 was 18,613 individuals [38-40].

### Measures

Access to mobile technology was measured by Internet use and cell phone ownership. Internet use was measured by the question: “Have you accessed the Internet in the past month?” Cell phone use was measured by the question: “Do you own a cell phone?” Both measures were dichotomized into yes–no answers.

#### Demographic Characteristics

Demographic characteristics included age, gender, education, marital status, living arrangement, rural/urban residence (household registration or Hukou), employment status, and income. In this study, education was categorized into four groups: primary school or less, middle school, high school or vocational school, and some college or more. Living arrangement had three mutually exclusive categories: empty nester (living alone or with spouse), living with children, and living with someone other than children [41]. In terms of income, given that most rural residents have no regular income, expenditure is a better welfare measure than income in developing countries [42]. Thus in this study, we followed the other published studies from CHARLS and used per capita expenditure (PCE) of the past year in the household for income measure; PCE was log-transformed because of its skewness of distribution for analyzing its relationship with other variables [43-45].

#### Community Resources

Community resources were measured using the community-level survey conducted in 2011, which asked informed officials or personnel in the community about the characteristics of the communities in which the CHARLS respondents resided. Three measures of community resources were taken in this study: neighborhood amenities, health care facilities, and community organizations. Neighborhood amenities is a composite measure derived by summing the following services in the neighborhood: drinking water used (tap water, well, and river/spring), types of cooking fuel (gas, coal, and hay), waste disposal (moved away by truck, buried in village, burned, dumped into nearby river, or no management), and main toilet system (in-house, out-house, or open air; and for each type with or without flushing water). A composite score was created with a range of 0 to 14, with a higher score indicating more urbanized neighborhood amenities [41]. Health care facilities were measured by summing the number of health care facilities in the respondent’s neighborhood, including the general hospital, specialized hospital, Chinese medicine hospital, community health center, township hospital, health care post, village clinic, private clinic, and pharmacy. A composite score ranging from 0 to 8 was created, with higher values indicating more health care resources [41]. Community resources is a composite measure derived by summing the following facilities in the community: basketball playground, swimming pool, outside exercise facilities, other outdoor sports facilities, room for card games and chess games, room for Ping-Pong, calligraphy and painting club, dancing team or other exercise clubs, other entertainment facilities, organizations for helping the elderly and the handicapped, activity center for residents, and an association for elders. A score ranging from 0 to 12 was created, with a higher value indicating more community resources [44].

#### Geographic Areas

Geographic areas were measured by the location where the survey was taken to capture the vast geographic differences in economic development and health care resources in China [45]. Three major regions of East China, Central China, and West China were included [46].

#### Health Outcomes

Health outcomes were measured by two indicators: self-reported health and having a disability. Such an approach allowed us to gauge older adults’ perceptions of their health, both generally and specifically, in relation to performing daily activities [45]. Self-reported health was a subjective measure of one’s health and was reported on the following scale: very good, good, fair, poor, or very poor. The responses were dichotomized to having good health (good or very good) and poor health (fair, not good, or poor) [43]. Having a disability was measured by two scales: the 6-item scale of activities of daily living (ADL) such as dressing and bathing and the 5-item scale of instrumental activities of daily living (IADL) such as preparing meals and taking medications. These 11 items were dichotomously coded (yes–no); no disability was defined as having no difficulty in all ADL or IADL items [43,46].

### Data Analysis

First, we used the chi-square (for categorical variables) and *t*-test (for continuous variables) to examine the relationship between access to mobile tools and individual-level SES, community resources, health outcomes, and geographic regions. Second, because the CHARLS dataset has a natural hierarchical structure with individuals nested within the community and the aim of this paper was to analyze the effects of individual and community characteristics on the digital divide and health disparities, we used two-level mixed logistic regression, controlling for unobserved heterogeneity at the community and individual levels to explore the relationship between access to mobile tools and SES, community resources, and geographic regions [41,44,45]. Odds ratios (ORs) with CI were employed to depict the relationship between outcome variable and independent variable while controlling for other covariates. Finally, to examine the cross-sectional relationship between health outcomes and access to mobile tools, we made four separate multivariate logistic regression models for two dependent variables of health outcomes (self-reported health and having a disability) and two independent variables of interest (Internet access and mobile phone ownership) while controlling for potential confounders of SES, community resources, and geographic region. All analyses, both descriptive tables and regressions, were weighted using individual sampling weights with household and individual nonresponse adjusted. Sixteen variables with missing data were multiple imputed by the Windows software NORM [47]. All analyses were performed using Stata 13 (StataCorp).

## Results

### Participant Characteristics: Socioeconomic Status, Health Outcomes, and Community Resources

Of the 18,215 participants included in this study, 44% of the participants were from East China, 26% from Central China, and 30% from West China. The mean age was 61 years, and 51% of the participants were female. Approximately 62% of the participants had a primary school education or less, 22% had completed middle school, 13% had high school or vocational school education, and 3% had some college education or had completed college. More than 84% of the participants were married; about 59% of the participants lived alone or with a spouse, 6% lived with children, and 35% lived with others. About 70% of the participants were rural residents, and 39% were unemployed. The average PCE was 15,914.78 Chinese Yuan (or US $2316.80) per year. More than 26% of the participants reported having poor health and about 40% reported having a disability. The score for the average number of neighborhood amenities was 7.10 (range: 0-12); the mean number of health care facilities nearby was 1.37 (range: 0-6), and community resources was 4.40 (range: 0-14).

### Binary Relationship of Access to Mobile Tools and SES, Health Outcomes, Community Resources, and Geographic Region

About 6.5% of the middle aged and elderly Chinese used the Internet, and 83% owned a mobile phone. As shown in Table 1, Internet access was associated with most of the variables of SES (except employment status), health outcomes, community resources, and geographic region. Specifically, significant age differences (54 years vs 61 years) existed between those who accessed Internet and those who did not. More males than females used the Internet (7.8% vs 5.3%), and Internet use was also associated with higher levels of education, being married, living with children, and urban residence; however, it was not related to employment status. Internet access was also associated with having good health and no disability. Three indicators of community resources were also significantly associated with Internet access, and so was geographic region. Similar patterns were observed in the binary relationship between mobile phone ownership and SES, health outcomes, community resources, and geographic region, with the exception of community organization.

**Table 1:** Relationship between the use of mobile technology (Internet use and mobile phone) and sociodemographic characteristics, community amenities, and geographic location (weighted).

Variables		Mean or category	Total	Internet use				Mobile phone use				
				Yes	No	χ^2^(df) or *t* (df)	*P*		Yes	No	χ^2^(df) or *t* (df)	*P*
**Socioeconomic status**												
	Age, in years	Mean	60.54	54.07	60.99	−13.7 (17897)	<.001		58.88	68.70	−26.3 (17897)	<.001
	Gender, n (%)	Female	9366 (51.42)	501 (5.35)	8865 (94.65)	43.2 (1)	.009		7696 (82.17)	1670 (17.83)	12.822 (1)	.04
		Male	8849 (48.58)	688 (7.78)	8160 (92.22)				7449 (84.18)	1400 (15.82)		
	Education, n (%)	≤Primary school	11,224 (61.62)	156 (1.39)	11,068 (98.61)	2428.3 (3)	<.001		8910 (79.38)	2314 (20.62)	296.423 (3)	<.001
		≤Middle school	4029 (22.12)	268 (6.64)	3762 (93.36)				3589 (89.07)	440 (10.93)		
		≤High school/ Vocational school	2375 (13.04)	538 (22.67)	1837 (77.33)				2135 (89.89)	240 (10.11)		
		≥College	587 (3.22)	224 (38.25)	362 (61.75)				516 (87.92)	71 (12.08)		
	Marital status, n (%)	Unmarried	2805 (15.40)	109 (3.88)	2696 (96.12)	37.1 (1)	.04		1779 (63.41)	1026 (36.59)	908.9 (1)	<.001
		Married	15,410 (84.60)	1077 (6.99)	14,333 (93.01)				13370 (86.76)	2040 (13.24)		
	Living arrangement, n (%)	Empty nest (alone or with spouse)	10,730 (58.91)	931 (8.68)	9799 (91.32)	279.0 (2)	<.001		8690 (80.98)	2041 (19.02)	89.9 (2)	<.001
		Living with children	1186 (6.51)	112 (9.41)	1074 (90.59)				1041 (87.81)	145 (12.19)		
		Living with others	6299 (34.58)	144 (2.28)	6155 (97.72)				5418 (86.02)	881 (13.98)		
	Rural-urban residence, n (%)	Urban	5554 (30.49)	984 (17.71)	4570 (82.29)	1615.5 (1)	<.001		4753 (85.59)	800 (14.41)	32.8 (1)	.008
		Rural	12,661 (69.51)	203 (1.60)	12,459 (98.40)				10396 (82.11)	2265 (17.89)		
	Employment status, n (%)	Not	7095 (38.95)	480 (6.77)	6614 (93.23)	1.2 (1)	.68		5325 (75.05)	1770 (24.95)	537.2 (1)	<.001
		Yes	11,120 (61.05)	706 (6.35)	10,414 (93.65)				9824 (88.34)	1297 (11.66)		
	Income (PCE^a^), in Chinese Yuan	Mean	15,914.78	33,953.60	14,657.84	7.9 (17897)	<.001		16,511.92	12,964.29	5.6 (17897)	<.001
**Health outcomes**												
	Self-reported health, n (%)	Poor	4663 (25.60)	125 (2.68)	4538 (97.32)	148.8 (1)	<.001		3694 (79.22)	969 (20.78)	68.5	<.001
		Good	13,552 (74.40)	1061 (7.83)	12,491 (92.17)				11,455 (84.53)	2096 (15.47)		
	Having a disability, n (%)	No	10,993 (60.35)	846 (7.70)	10,146 (92.3)	54.5 (1)	.003		9453 (85.99)	1540 (14.01)	110.6 (1)	<.001
		Yes	7222 (39.65)	352 (4.88)	6870 (95.12)				5779 (80.01)	1444 (19.99)		
											
**Community resources**												
	Neighborhood amenities^b,c^	Mean	7.10	10.83	8.84	34.4 (17897)	<.001		7.15	6.9	1.8 (17897)	.08
	Health care facilities^c,d^	Mean	1.37	1.79	1.34	5.3 (17897)	<.001		1.38	1.30	2.3 (17897)	.02
	Community organizations^c,e^	Mean	4.40	7.14	4.21	14.2 (17897)	<.001		4.38	4.50	−0.7 (17897)	.47
**Region**												
	East, n (%)		8058 (44.24)	728 (9.03)	7331 (90.97)	168.9 (2)	<.001		6713 (83.3)	1346 (16.70)	32.9 (1)	.003
	Central, n (%)		4718 (25.90)	270 (5.73)	4447 (94.27)				3810 (80.76)	908 (19.24)		
	West, n (%)		5439 (29.86)	188 (3.46)	5251 (96.54)				4626 (85.06)	813 (14.94)		

^a^PCE: Per capita expenditure.

^b^Neighborhood amenities comprised four variables (tap water, toilet, cooking fuel, and waste management); a range of 0 to 14, with higher value meaning higher coverage of modern amenities.

^c^Community/neighborhood data were not available for 2013; it was only available in 2011.

^d^Health facilities include six variables such as clinic, pharmacy, and hospital; with a range of 0 to 8.

^e^Community organizations include 14 variables such as having a senior activity room, having a community council, having a playground; a range of 0 to 12, with higher value meaning more community resources.

### Multivariate Relationship of Access to Mobile Tools and SES, Community Resources, and Geographic Region

After controlling for potential confounders, Internet use was independently and significantly associated with the following predictors: age, gender, education level, marital status, living arrangement, rural/urban residency, income, neighborhood amenities, and geographic region, but it was not associated with employment status, health care facilities, and community organizations (see Table 2). Likewise, mobile phone ownership was independently and significantly associated with age, education level, marital status, living arrangement, rural/urban residency, income, and geographic region, but it was not associated with gender, employment status, neighborhood amenities, health care facilities, and community resources.

### Multivariate Relationship of Access to Mobile Tools and Health Outcomes

Table 3 depicts the results of four models on the relationship of access to mobile tools and health outcomes. Internet access was significantly associated with self-reported health (adjusted odds ratio, aOR=1.73) but not related to disability while controlling for potential confounders. Mobile phone ownership was significantly associated with disability (aOR=0.843) but not with self-reported health. Other predictors of good self-reported health included gender, education, rural/urban residency, employment status, neighborhood amenities, and geographic region. Other predictors of having a disability included age, education, marital status, living arrangement, rural/urban residency, employment status, neighborhood amenities, community organization, and geographic region. We also analyzed the data by using health outcomes as continuous variables, and the results were similar.

**Table 2:** Estimates of fixed and random parameters from the multilevel mixed models of mobile technology use (Internet, mobile phone) on socioeconomic status, neighborhood amenities, and community resources (weighted).

Variables				Internet use				Mobile phone		
				aOR (95% CI)		*P*		aOR (95% CI)		*P*
Intercept				0.007 (0.001-0.044)		<.001		12.980 (4.733-35.600)		<.001
**Individual-level variables**										
	Mean age, in years			0.912 (0.897-0.928)		<.001		0.928 (0.921-0.936)		<.001
	Gender (Ref=female)			1.436 (1.207-1.708)		<.001		1.059 (0.989-1.134)		.10
	**Education (Ref=≤primary school)**									
		≤Middle school	3.951 (2.932-5.326)		<.001		1.238 (1.076-1.423)		.003	
		≤High school/Vocational school	9.409 (7.091-12.49)		<.001		1.336 (1.099-1.624)		.004	
		≥College	20.24 (13.90-29.45)		<.001		1.020 (0.673-1.547)		.93	
	Marital status (Ref=unmarried)			0.858 (0.592-1.243)		.42		1.948 (1.668-2.276)		<.001
	**Living arrangement (Ref=empty-nested)**									
		Living with children	0.888 (0.538-1.464)		.64		2.193 (1.634-2.943)		<.001	
	** **	Living with others	0.886 (0.679-1.157)		.37		2.269 (1.970-2.614)		<.001	
	Rural-urban residence (Ref=urban)			0.368 (0.279-0.485)		<.001		0.553 (0.455-0.672)		<.001
	Employment status (Ref=unemployed)			1.047 (0.823-1.332)		.71		1.505 (1.328-1.706)		<.001
	Log income, mean (per capita expenditure)			1.593 (1.407-1.802)		<.001		1.441 (1.340-1.549)		<.001
**Community-level variables**										
	**Community resources**									
		Neighborhood amenities, mean	1.188 (1.127-1.253)		<.001		1.008 (0.977-1.040)		.62	
		Health facilities, mean	1.037 (0.924-1.164)		.54		0.995 (0.914-1.082)		.89	
		Community organization, mean	1.018 (0.976-1.063)		.41		0.960 (0.926-0.994)		.02	
	**Region (Ref=east)**									
		Central	1.328 (0.965-1.828)		.08		0.875 (0.707-1.083)		.22	
		West	0.755 (0.534-1.069)		.11		1.259 (1.020-1.553)		.03	
Random effect variance				1.734 (1.420-2.116)		<.001		1.741 (1.527-1.984)		<.001

**Table 3:** Estimates of fixed and random parameters from the multilevel mixed models of health outcomes and mobile technology use (weighted).

Variables			Internet access as primary predictor				Mobile phone ownership as primary predictor			
			Self-reported health		Having a disability		Self-reported health		Having a disability	
			aOR (95% CI)	*P*	aOR (95% CI)	*P*	aOR (95% CI)	*P*	aOR (95% CI)	*P*
Intercept			2.421 (1.330-4.406)	.004	0.193 (0.114-0.328)	<.001	2.298 (1.245-4.240)	.008	0.229 (0.133-0.392)	<.001
Internet use (Ref=no Internet use)			1.727 (1.327-2.246)	<.001	1.138 (0.945-1.371)	.17	—^a^		—^a^	
Own mobile phone (Ref=no mobile phone)			—^b^		—^b^		1.076 (0.968-1.196)	.17	0.843 (0.763-0.931)	<.001
**Individual-level variables**										
	Age		0.988 (0.983-0.992)	<.001	1.022 (1.018-1.026)	<.001	0.988 (0.983-0.992)	<.001	1.020 (1.015-1.024)	<.001
	Gender (Ref=female)		1.373 (1.278-1.475)	<.001	1.010 (0.944-1.080)	.77	1.374 (1.279-1.476)	<.001	1.012 (0.946-1.082)	.74
	**Education (Ref=≤primary school)**									
		≤Middle school	1.121 (1.023-1.228)	.01	0.874 (0.808-0.945)	<.001	1.129 (1.031-1.236)	.009	0.878 (0.812-0.949)	.001
		≤High school/Vocational school	1.282 (1.110-1.481)	<.001	0.890 (0.792-1.001)	.05	1.333 (1.155-1.538)	<.001	0.905 (0.807-1.016)	.09
		≥College	1.486 (1.099-2.010)	.01	0.887 (0.675-1.166)	.39	1.659 (1.228-2.239)	<.001	0.917 (0.703-1.196)	.52
	Marital status (Ref=unmarried)		0.913 (0.816-1.022)	.11	0.827 (0.742-0.922)	<.001	0.902 (0.805-1.010)	.07	0.842 (0.755-0.938)	.002
	**Living arrangement (Ref=empty nested)**									
		Living with children	1.047 (0.875-1.252)	.62	1.114 (0.967-1.285)	.14	1.038 (0.866-1.243)	.07	1.129 (0.978-1.304)	.09
		Living with others	1.139 (1.054-1.232)	.001	0.951 (0.885-1.023)	.02	1.128 (1.041-1.221)	.003	0.965 (0.897-1.038)	.34
	Rural/urban residence (Ref=urban)		0.731 (0.648-0.824)	<.001	1.295 (1.167-1.437)	<.001	0.722 (0.640-0.814)	<.001	1.273 (1.148-1.413)	<.001
	Employment status (Ref=unemployed)		2.239 (2.044-2.454)	<.001	0.619 (0.568-0.674)	<.001	2.235 (2.040-2.449)	<.001	0.623 (0.572-0.679)	<.001
	Log income, mean (per capita expenditure)		1.01 (0.969-1.051)	.64	1.028 (0.991-1.067)	.13	1.011 (0.971-1.052)	.60	1.037 (0.999-1.076)	.05
**Community-level variables**										
	**Community resources**									
		Neighborhood amenities, mean	1.074 (1.055-1.093)	<.001	0.976 (0.961-0.990)	.001	1.076 (1.057-1.095)	<.001	0.976 (0.962-0.991)	.002
		Health facilities, mean	0.997 (0.946-1.051)	.90	1.008 (0.964-1.055)	.72	0.997 (0.946-1.051)	.91	1.008 (0.964-1.055)	.72
		Community organization, mean	1.017 (0.998-1.035)	.07	0.982 (0.967-0.998)	.03	1.017 (0.999-1.036)	.06	0.982 (0.966-0.998)	.02
	**Region (Ref=east)**									
		Central	0.878 (0.772-0.999)	.05	1.272 (1.137-1.422)	<.001	0.883 (0.775-1.005)	.06	1.270 (1.135-1.422)	<.001
		West	0.775 (0.687-0.875)	<.001	1.541 (1.382-1.718)	<.001	0.772 (0.684-0.871)	<.001	1.547 (1.387-1.726)	<.001
Random effect variance			1.120 (1.078-1.163)	<.001	1.102 (1.070-1.135)	<.001	1.120 (1.078-1.163)	<.001	1.104 (1.072-1.138)	<.001

^a^Mobile phone ownership as primary predictor, the parameters of Internet use are missing.

^b^Internet access as primary predictor, the parameters of mobile phone ownership are missing.

## Discussion

### Principal Findings

Our data analysis revealed that only a small percentage (6.5%) of middle-aged and elderly Chinese participants accessed the Internet, but a high proportion (83%) of the participants owned a mobile phone. The rate of access to the Internet was much lower than that of the official report of Internet access in the general population (53%; [25]). Such a discrepancy might be because of three possible reasons. First, our measure of Internet access was based on the question—“Have you accessed Internet in the past month?,” but the other surveys on Internet use typically measured lifetime use. Second, many older adults interpret “accessed Internet” to mean going online via a computer only; many have used mobile phones for Web-based activities but did not report so. Third, prior studies have relied on voluntary convenience sampling, and middle-aged and elderly people and rural residents were less likely to be included in the survey; however, the rate of mobile phone ownership was comparable with that of general population (95%; [26]).

Our findings corroborated existing literature on the relationship of SES and digital divide by adding new evidence from middle-aged and elderly Chinese. Similar to other countries, access to mobile tools was associated with younger age, higher level of education, higher income, and urban residence [4,8,11,12,17,20,22]. Our data also suggested different sets of SES predictors for Internet access and mobile phone ownership; for example, significantly fewer women than men accessed Internet, but gender was not a significant predictor of mobile phone ownership. The high rate of mobile phone ownership in China may provide an equal opportunity for women to access information [48]. By contrast, people who were married or living with children or others were more likely to own mobile phones compared with people who are single or living alone, but Internet access was not associated with marital status or living arrangement. This may suggest that the mobile phone has instead become an important communication tool for people living with families or others.

Our study also examined the effect of community-level SES on access to mobile tools. We found that Internet access was strongly associated with neighborhood amenities (drinking water, toilet, etc) but not health care facilities or community organization; mobile phone ownership was not associated with any of the three measures of community resources. Neighborhood amenities were a good indicator of urbanization [43], and their association with Internet access suggests that Internet access may be considered a kind of community resource. Because urban areas are better equipped with broadband access, urban residents are more likely to access the Internet. The lack of a relationship between mobile phone ownership and community resources suggests that mobile phone as a portable and personal communication tool has wider accessibility, as it is less likely to be restricted by community-level facilities or resources.

In the analysis on the relationship of the digital divide and health disparities, our data showed that self-reported health was significantly associated with Internet use, and mobile phone ownership was significantly associated with not having a disability. Such findings were consistent with the literature on the relationship of Internet access and status [3,13]. As documented in the literature and also described above, Internet access and mobile phone ownership were important indicators of SES at individual and community levels; therefore, the relationship between Internet access and health status was de facto the reciprocal relationship of SES and health outcomes [4,10,17].

We have also observed significant health disparities in our participants; health disparities were predicted by SES, rural/urban residence, community resources, and geographic region, which were consistent with prior CHARLS studies [41,43-46]. Such disparities reflect the inequality of resource allocation and economic development in rural and urban areas and across regions.

The following policy implications are associated with the forgoing empirical findings on the digital divide and health disparities in China. First, very few middle-aged and elderly Chinese use the Internet, which is a strong predictor of SES and health outcomes. Because community SES has been recognized as a strong predictor of individual health [16], building community resources has been advocated as an important strategy to improve health [43,44]. China promotes the “Internet of Things” and “intelligent hospitals” [27-29], so improving access to mobile tools, especially the Internet for underserved communities and in underdeveloped areas, would potentially yield significant improvement in health outcomes.

Second, a majority of older Chinese have owned a mobile phone. The high ownership rate of the mobile phone suggests that it could become a tool that transcends social classes and reaches the vulnerable and underserved. If welfare covers food and rent as necessities of life, vouchers for mobile phone subscriptions could also be considered so that the elderly and people with a disability or those who are living alone could have access to this basic communication tool [8,10]. Existing literature has shown that closing the digital gap is conducive to bridging health [49].

Third, as more people are connected with mobile phones, such ubiquitous access can be maximized upon for empowerment and health services delivery. Researchers have documented initial evidence of the efficacy of mobile phone–delivered health intervention [50]; however, most of these mobile health (mHealth) interventions were conducted in Western countries with limited data from developing countries such as China, despite a high ownership rate of mobile phones. In recent years, some scientists have piloted mobile-based interventions [36,37,51-53]. For example, a recent study showed that text messages could effectively promote smoking cessation in Chinese adults [37]. Such endeavors would be especially beneficial for the elderly Chinese, given that China is aging rapidly, thanks to its improved life expectancy and the decline in fertility. The three-decade one-child policy has dramatically affected the elderly care model in China, and the current resources cannot keep up with the rapidly growing aging population [54]. Some experts have called for innovative mHealth solutions for chronic condition management and elderly care in China [55]. Widely accessible mobile phones and continuing penetration of Internet access may be a part of the solutions.

### Strengths and Limitations

Our study has the following strengths. First, it was based on a national probability sample of older adults in China and had a large sample size; therefore, our findings can be generalized to other older adults in China. Second, the SES measures included variables at individual, household, and community levels, thus giving us a comprehensive measurement of SES. Third, our health outcomes were measured by both self-reported health and disability using two scales and 11 items relevant to older adults in China.

We also note the following limitations in our study. First, the study design was cross-sectional in nature, and we could infer no causal relationship. In addition, the community resources were collected in 2011 (data was not available in 2013), but health outcomes, access to mobile tools, and other covariates were collected in 2013. The relationship of community resources and access to the Internet and health outcomes may be predictive. Second, the CHARLS includes only 2 simple questions on access to mobile tools, so we could measure only Internet use and mobile phone ownership. Other important aspects of mobile tool use, including the length of use, frequency of use, purpose of use, and whether they use smartphones, were missing in this study; however, a recent survey has shown that smartphone users in China accounted for 53% of all mobile phone users in 2016 [56]. Whether and how people use mobile tools for health purposes affects the relationship of the digital divide and health disparities [57]; therefore, we call for more data on access to and usage of mobile tools. Third, our measures of health outcomes were also limited to two indicators. Furthermore, the dichotomous nature of the health outcome variables may limit our analysis of the relationship of the digital divide and health disparities.

### Conclusions

To conclude, to the best of our knowledge, this study is the first on the digital divide and health disparities in China. Our study advanced the literature by providing data on the relationship of SES and the digital divide by embedding individual characteristics in community resources. A low rate of Internet access and its strong relationship with neighborhood amenities and health outcomes suggest that Internet access may be an important indicator of individual- and community-level SES, and more Internet access may result in upgrades in individual SES and community infrastructure. By contrast, the high ownership rate of mobile phone and its lack of relationship with community resources suggest that the mobile phone may transcend social classes and become an ordinary personal item. The high ownership of mobile phones presents an enormous potential to empower and serve older Chinese, who face mounting challenges in care in a rapidly aging society. We call for more studies on the use of mobile tools and its relationship with health disparities in China.

## Abbreviations

ADL: 6-item scale of activities of daily living
aOR: adjusted odds ratio
CAPI: computer-aided personal interviews
CHARLS: China Health and Retirement Longitudinal Study
eHealth: electronic health
GDP: gross domestic product
HRS: Health and Retirement Study
IADL: 5-item scale of instrumental activities of daily living
mHealth: mobile health
OR: odds ratio
PCE: per capita expenditure
PPS: probability proportional to size
SES: socioeconomic status
SHARE: Survey of Health and Retirement in Europe
